# Genetic and clinical characteristics of Chinese children with Glucokinase-maturity-onset diabetes of the young (GCK-MODY)

**DOI:** 10.1186/s12887-018-1060-8

**Published:** 2018-03-06

**Authors:** Xiuzhen Li, Tzer Hwu Ting, Huiying Sheng, Cui Li Liang, Yongxian Shao, Minyan Jiang, Aijing Xu, Yunting Lin, Li Liu

**Affiliations:** 1 Department of Genetics and Endocrinology, Guangzhou Women and Children’s Medical Center, 9 Jinsui Road, Guangzhou, Guangdong 510623 China; 2 Department of Paediatrics, Faculty of Medicine & Health Sciences, University Putra Malaysia, 43400 Serdang, Selangor Malaysia

**Keywords:** MODY, Glucokinase, Genetics, Chinese, Children

## Abstract

**Background:**

There is scarcity of information on the clinical features and genetics of glucokinase-maturity-onset diabetes of the young (GCK-MODY) in China. The aim of the study was to investigate the clinical and molecular characteristics of Chinese children with GCK-MODY.

**Methods:**

Eleven children with asymptomatic hyperglycemia and clinically suspected GCK-MODY were identified from the database of children with diabetes in the biggest children’s hospital in South China. Clinical data were obtained from medical records. Blood was collected from the patients and their parents for glucokinase (GCK) gene analysis. Parents without diabetes were tested for fasting glucose and HbA1c. Clinical information and blood for GCK gene analysis were obtained from grandparents with diabetes. GCK gene mutational analysis was performed by polymerase chain reaction and direct sequencing. Patients without a GCK gene mutation were screened by targeted next-generation sequencing (NGS) technology for other MODY genes.

**Results:**

Nine children tested positive for *GCK* gene mutations while two were negative. The nine GCK-MODY patients were from unrelated families, aged 1 month to 9 years and 1 month at first detection of hyperglycaemia. Fasting glucose was elevated (6.1–8.5 mmol/L), HbA1c 5.2–6.7% (33.3–49.7 mmol/mol), both remained stable on follow-up over 9 months to 5 years. Five detected mutations had been previously reported: p.Val182Met, c.679 + 1G > A, p.Gly295Ser, p.Arg191Gln and p.Met41Thr. Four mutations were novel: c.483 + 2 T > A, p.Ser151del, p.Met57GlyfsX29 and p.Val374_Ala377del. No mutations were identified in the other two patients, who were also tested by NGS.

**Conclusions:**

*GCK* gene mutations are detected in Chinese children and their family members with typical clinical features of GCK-MODY. Four novel mutations are detected.

## Background

Maturity-onset diabetes of the young (MODY) is a rare form of diabetes mellitus with autosomal dominant inheritance, typically diagnosed before 25 years of age. It is caused by mutations in 13 genes involved in pancreatic β-cell function [1]. One of the most common types reported is MODY2, which is caused by heterogenous inactivating mutations in the glucokinase (*GCK*) gene, hence also known as glucokinase-maturity-onset diabetes of the young (GCK-MODY) [1].

GCK-MODY is characterized by asymptomatic fasting hyperglycaemia, which is present from birth and remains stable throughout life [2]. Typically the fasting glucose falls in the range 5.6–8.0 mmol/L with hemoglobin A1c (HbA1c) 5.6–7.3% (38–56 mmol/mol) in those aged 40 years and below, as shown in a large cohort of GCK-MODY patients [3].

Glucokinase (GCK) is a key enzyme in the regulation of insulin release in pancreatic β-cells. It is encoded by the glucokinase gene located at chromosome 7p15.3–p15.1, consisting of 10 exons and spanning 45,168 bp [4]. Abnormalities in GCK due to gene mutations will disrupt glucose homeostasis causing both hyperglycaemia and hypoglycaemia. Heterozygous inactivating mutations cause GCK-MODY while homozygous or compound heterozygous mutations result in a more severe phenotype of permanent neonatal diabetes mellitus. In contrast, heterozygous activating mutations cause persistent hyperinsulinemic hypoglycaemia of infancy [4].

There are 620 different mutations found in 1441 families in the 10 exons (exons 1–10) of the *GCK* gene expressed in the pancreatic β-cells [4]. Missense, nonsense, frameshift, and splice site mutations are commonly reported and most of the mutations are private [4]. Although heterozygous pathogenic *GCK* mutations are diverse, they all lead to the same phenotype of mild fasting hyperglycaemia.

Most of the studies on GCK-MODY are in Caucasian populations. Studies on *GCK* mutations in Asian populations are few and showed a much lower frequency of mutations (less than 5%) in those with clinical diagnosis of MODY [5–7]. However, a more recent study involving 80 patients in Japan revealed a higher frequency of *GCK* mutations in 22.8% of paediatric-onset MODY patients, comparable to European studies [8]. An Indian study found a low frequency of *GCK* mutations in children and adolescents with mild hyperglycaemia [9]. As GCK-MODY studies are scarce in Chinese populations, little is known about the clinical features of GCK-MODY in Chinese children. We previously reported a boy who was misdiagnosed as type 1 diabetes and was treated with insulin for half a year until he was identified to be GCK-MODY [10]. We therefore realized that many patients with GCK-MODY might be missed and misdiagnosed as other types of diabetes. In an attempt to improve the diagnosis and management of GCK-MODY in China, we investigated the clinical and molecular characteristics of Chinese children with a clinical suspicion of GCK-MODY seen in the biggest children’s hospital in South China.

## Methods

### Patients

From April 2011 to April 2016, there were 587 children with newly diagnosed diabetes mellitus in Guangzhou Women and Children’s Medical Center (the biggest children’s hospital in South China). There were 24 children with clinically diagnosed MODY. Eleven of these 24 children had asymptomatic hyperglycaemia and were clinically suspected to have GCK-MODY. *GCK* gene mutation analysis was performed in these eleven children.

The presumptive clinical diagnosis of GCK-MODY was established by the following features according to the European guidelines [11, 12]: 1) asymptomatic fasting hyperglycaemia ≥5.5 mmol/L, HbA1c < 7.5% (59 mmol/mol), and small 2-h glucose increment < 3 mmol/L in an oral glucose tolerance test (OGTT). 2) parents have diabetes without complications or unaffected parents have mild fasting hyperglycaemia 5.5–8 mmol/L.

Details of clinical data were obtained from medical records. Clinical follow-up started from the time of diagnosis and subsequently at 3–6 month intervals. Self monitored blood glucose levels were recorded, and HbA1c, height and weight were measured at every visit. OGTT was performed yearly with an oral glucose dose of 1.75 g/kg body weight (maximum 75 g) after a minimum of 8 h of fasting.

Parents without history of diabetes mellitus were tested for fasting plasma glucose (FPG) and HbA1c. Clinical information and recent FPG and HbA1c were obtained from parents and grandparents with a history of diabetes.

Informed consent was obtained from all patients’ parents. The study was approved by the Institutional Review Board of Guangzhou Women and Children’s Medical Center. The mutational analysis of *GCK* gene was performed at Guangzhou Women and Children’s Medical Center from May 2012 to May 2016.

### Laboratory evaluation

The following biochemical parameters were measured on patients blood samples: plasma glucose by enzymatic method; HbA1c by latex immunoagglutination inhibition methodology (DCA Systems, Siemens, Erlangen, Germany); serum fasting insulin by chemiluminescence immunoassay (ADVIA Centaur XP Immunoassay Systems, Siemens, Erlangen, Germany); anti-glutamic acid decarboxylase (GAD), anti-islet cell (ICA) and anti-insulin (IAA) antibodies by radioimmunoassay.

### Mutational analysis of *GCK* gene and other genes associated with monogenic diabetes

Genomic DNA was extracted from peripheral blood leukocytes of the probands, their parents and grandparents, using a whole blood DNA extraction kit (Qiagen 51,106 QIAamp DNA Mini Kit, Germany) according to the manufacturer’s protocol. Exons 1–10 and exon-intron boundaries of the GCK gene were amplified by polymerase chain reaction (PCR) (Mastercyclers Pro TM Gradient Thermal Cycler, Eppendorf, Hamburg, Germany). The primer sequences are listed in Table 1. DNA sequence analyses were conducted with DNA Analyzer 3730(ABI, USA). Sequences were compared with the reference sequence (NM_000162.3) using DNAMAN and Chromas software (V.2.01, Technelysium Pty Ltd., Tewantin QLD, Australia). The novel mutations in the study were determined by comparing with the SNP databases, including 1000Genomes, ESP6500, ExAc and dbSNP, and the Human Gene Mutation Database (HGMD). The pathogenicity of the mutation occurring in flanking intronic regions resulted in abnormal splicing was predicted by the online tools of MutationTaster, NetGene2, and Human Splicing Finder V3.0, while only MutationTaster was used for frame shift or deletion type mutations.

**Table 1:** Primers for amplification of Glucokinase (GCK) gene coding sequences

Exons	Primers	Sequence (5′-3′)	Annealing temperature (°C)
Exon1	1F	ATTTCCACTTCAGAAGCCTACT	58
	1R	GGCTCAAACAAACCATGGAAT	
Exon2	2F	GGGGTCAGAAGACAGAAGGAGGC	64
	2R	TGAGAACTGGCCCAAGTCGAGGA	
Exon3	3F	GTAATATCCGGGCTCAGTCACC	64
	3R	ACAGGTGGCACCTCCCGTCAG	
Exon4	4F	ATAGCTTGGCTTGAGGCCGTG	62
	4R	TTTGAAGGCAGAGTTCCTCTG	
Exon5–6	5–6F	CTGCTCTGAGCCTGTTTCC	62
	5–6R	ACGGTGCTTCCATCTTGAT	
Exon7	7F	CCGCCTTTCCATTGTTCC	64
	7R	CTCCCATCTGCCGCTGCACC	
Exon8	8F	AGGAAGGTTTCGGAGGGACT	62
	8R	TGAGACCAAGTCTGCAGTGCC	
Exon9	9F	GATGGACTGTCGGAGCGACACT	64
	9R	TCTTGGAGCTTGGGAACCGC	
Exon10	10F	AAGGGTCGACTGCGTGCAG	64
	10R	ATTCCAGCGAGAAAGGTG	

*F* forward, *R* reverse

2 patients in whom *GCK* mutations were not detected, were screened by targeted next-generation sequencing (NGS) technology. A capture panel (NimbleGen, Madison, USA) of monogenic diabetes genes was designed which comprised 157,624 bp that covered all exons together with the flanking exon and intron boundaries (±15 bp) of 44 genes, including *GCK*, *INS*, *HNF1A*, *HNF1B*, *HNF4A*, *KLF11*,*BLK*,*CEL*, *NEUROD1*, *NEUROD3*, *PDX1*, *KCNJ11*, *ABCC8*, *ZFP57*, *HYMAI*, *EIF2AK3*, *WFS1*, *AKT2*, *GLUD1*, *HADH*, *MAPK8IP1*, *PAX4*, *PLAGL1*, *PTF1A INHANCER*, *RFX6*, *SLC2A2*, *SLC19A2*, *UCP2*, *GLIS3*, *INSR*, *PTF1A*, *GATA6*, *IER3IP1*, *PAX4*, *FOXA2*, *SLC16A1*, *FOXP3*,*CISD2*, *CAPN10*, *PPAR*, *AGPAT2*, *BSCL2*, *IPF1* and *MNX1*. Then the genomic DNA samples was fragmented by Covaris LE220 (Massachusetts, USA) to generate a paired-end library (200–250 bp). The library was enriched by array hybridization according to the procedure described previously [13], followed by elution and post-capture amplification. The products were then subjected to Agilent 2100 Bioanalyzer to estimate the magnitude of enrichment. After quality control, captured library sequencing was carried out on Illumina HiSeq2500 Analyzers (Illumina, San Diego, USA) for 90 cycles per read to generate paired-end reads. Image analysis, error estimation, and base calling were performed using Illumina Pipeline software (version 1.3.4) to generate raw data with an average of 178-fold depth coverage to identify causal mutations.

## Results

Nine out of eleven children with asymptomatic hyperglycaemia were found to have heterozygous mutations in the *GCK* gene while two were negative for *GCK* gene mutations by direct sequencing. They were from eleven unrelated families from provinces of South China and were born to non-consanguineous parents. No mutations were identified in the other two patients, even after testing by NGS.

### Clinical features

Clinical data on each of the nine patients with mutations in the *GCK* gene are shown in Table 2. They were aged 1 month to 9 years and 1 month when hyperglycaemia was first detected. All were asymptomatic of hyperglycaemia and were otherwise well (except case 7, who had epilepsy at 2 years of age and was well controlled with levetiracetam treatment). They had unremarkable physical examinations without dysmorphic features or acanthosis nigricans. All had normal nutritional status. Pancreatic autoantibodies were negative in all patients. In eight patients (case1 and cases 3–9), FPG was elevated to 6.1–8.5 mmol/L and remained stable over time ranging from 9 months to 5 years without any medication. HbA1c of the nine patients at diagnosis ranged from 5.2–6.7% (33.3–49.7 mmol/mol), three of them (cases 7–9) had diabetic HbA1c ≥ 6.5% (47.5 mmol/mol). Five patients (case 1 and cases 3–6) had non-diabetic HbA1c at diagnosis and on follow-up without drug treatment. One patient (case 9) had diabetic range HbA1c both at diagnosis and after 4 years of follow-up. All patients had OGTT showing impaired glucose tolerance at diagnosis with 2-h glucose increment < 3 mmol/L except case 3 (3.1 mmol/L). One patient (case 9) had OGTT showing diabetes with 2-h glucose increment > 3 mmol/L (4.6 mmol/L) after 4 years of follow-up. Repeat OGTT on recent follow-up (duration ranging from 9 months to 5 years) in the others showed impaired glucose tolerance with 2-h glucose increment < 3 mmol/L in 6 patients while one (case 5) had 2-h glucose increment > 3 mmol/L (3.5 mmol/L).

**Table 2:** Clinical features of 9 patients with GCK-MODY from south China

Patients	Case 1	Case 2	Case 3	Case 4	Case5	Case 6	Case 7	Case 8	Case9
At diagnosis									
Age	1 m	3 m	1 m	6 y11 m	9 y1m	2 y9m	4y8m	8 y11m	8 y6 m
Gender	M	M	F	F	F	M	M	M	M
Gestational age(w + d)	38 + 1	37 + 6	34 + 6	38 + 4	39 + 1	38 + 6	37 + 5	37 + 5	38
Birth weight (kg)	2.7	2.7	1.4	3.5	3.5	3.3	2.6	3.15	2.5
Family history of diabetes	Father	Father/ Grandmother	Mother	Mother	Mother	Mother/ Grandfather	Father	Mother/ Grandmother	Father/ Grandfather
BMI percentile	50th	25th	No data	25th	50th	25th	75th	50th	50th
FPG (mmol/L)	8.5	7.2	6.5	6.8	6.1	7.1	7.3	7.1	7.2
OGTT 2hPG (mmol/L)	No data	8.0	9.6(at 3 m)	7.8	7.6	7.6	8.4	8.2	8.8
2hPG increment (mmol/L)	No data	0.8	3.1	1.0	1.5	0.5	1.1	1.1	1.6
HbA1c (%/mmol/mol)	5.8/39.9	5.2/33.3	4.6/26.8(at 3 m)	5.7/38.8	5.8/39.9	5.9/41.0	6.6/48.6	6.5/47.5	6.7/49.7
Fasting Ins (pmol/L)	30.5	29.7	13.1	30.9	31.5	24.9	15.1	7.8	23.5
DKA	no	no	no	no	no	no	no	no	no
Pancreatic autoantibody	negative	negative	negative	negative	negative	negative	negative	negative	negative
Urine sugar	negative	negative	negative	negative	negative	negative	negative	negative	negative
Other problems							EP at 2y		
Treatment and evolution	Diet, active exercise	Glibenclamide 0.3 mg/kg/d, stopped at 1y4m	Diet	Diet, active exercise	Diet, active exercise	Diet, active Exercise	Diet, active exercise	Diet, active Exercise	Diet, active exercise
Recent Follow-up									
Duration of follow-up	5 y	2 y 4 m	2 y1m	2 y6 m	1 y10 m	2 y	1 y1 m	9 m	4 y
Age	5 y1 m	2 y7m	2 y2 m	9 y5 m	10 y11 m	4 y9 m	5 y9 m	9y8 m	12 y6 m
BMI percentile	50th	25th	50th	50th	25th	25th	85th	50th	50th
HbA1c (%/mmol/mol)	6.0/42.1	6.4/46.4	6.1/43.2	5.8/39.9	5.9/41.0	6.0/42.1	6.4/46.4	6.5/47.5	7.5/58.4
FPG (mmol/L)	7.6	6.5	6.5	6.1	6.4	6.9	7.5	6.7	8.0
Fasting Ins (pmol/L)	13.9	10.4	0.99	8.6	14.9	16.6	5.9	9.2	6.7
OGTT 2hPG (mmol/L)	8.7	6.7	8.0	7.4	9.9	8.7	8.9	8.8	12.6
2hPG increment (mmol/L)	1.1	0.2	1.5	1.3	3.5	1.8	1.4	2.1	4.6
OGTT 2 h Ins (pmol/L)	90.6	19.4	23.8	43.9	61.4	47.9	32.9	49.6	52.1
Combined with other problems			Developmental delay				EP and MR, treated with levetiracetam		

*m* month, *y* year, *M* male, *F* female, *BMI* body mass index, *FPG* fasting plasma glucose, *PG* plasma glucose, *2 h* 2 h, *Ins* insulin, *DKA* diabetic ketoacidosis, *OGTT* oral glucose tolerance test, *EP* epilepsy, *MR* mental retardation

Case 2 was misdiagnosed as neonatal diabetes mellitus and received oral glibenclamide at the age of 3 months. At the age of 1 year 4 months, the medication was stopped when GCK-MODY was confirmed by genetic analysis. Both his FPG and HbA1c levels remained stable at 5.8–6.5 mmol/L and 6.2–6.7% (44.3–47.9 mmol/mol) respectively, after 1 year and 3 months of follow-up without medication. OGTT at age 2 years and 7 months showed impaired fasting glucose with normal 2-h glucose level.

All 9 patients had a parent with elevated FPG 5.8–7.8 mmol/L, and normal to mildly raised HbA1c 6.2–7.1% (44.3–54.1 mmol/mol) (Table 3).

**Table 3:** Clinical data of family members with diabetes mellitus or impaired fasting glucose

Patients	Family member	DM/IFG diagnosed age (years)	Treatment	At genetic confirmation of GCK-MODY			
				Age (years)	BMI (kg/m^2^)	FPG (mmol/L)	HbA1c (%/ mmol/mol)
Case 1	Father	32	No	37	20.4	6.5	7.1/54.1
Case 2	Father	35	No	38	19.5	7.1	6.9/51.9
	Grandmother	51	Glibenclamide	65	22.5	6.8	7.1/54.1
Case 3	Mother	–	–	29	20.5	6.4	6.8/50.8
Case 4	Mother	42	No	46	21.5	5.8	6.3/45.4
Case 5	Mother	39	Diet, active exercise	42	21.8	6.1	6.2/44.3
Case 6	Mother	–	–	35	20.9	7.8	6.4/46.5
	Grandfather	50	Metformin	70	22.7	6.3	6.5/47.5
Case 7	Father	–	–	37	24.1	6.2	6.4/46.5
Case 8	Mother	–	–	37	25.1	7.0	6.9/51.9
	Grandmother	42	Metformin for 2 years and wean off at age of 44 years	64	23.6	6.3	7.4/57.4
Case 9	Father	36	Metformin	39	21.8	6.9	7.0/53.0
	Grandfather	40	No	65	21.6	6.5	6.5/47.5

*DM* diabetes mellitus, *IFG* impaired fasting glucose, *BMI* body mass index, *FPG* fasting plasma glucose

### Sequencing analysis of the GCK gene

The *GCK* gene analysis identified 9 different heterozygous mutations in nine patients (Table 4). Five mutations were previously reported: c.544G > A (p.Val182Met), c.679 + 1G > A, c.883G > A (p.Gly295Ser), C.572G > A (p.Arg191Gln), and c.122 T > C (p.Met41Thr). Four mutations were novel and predicted to be deleterious using online bioinformatic tools: two deletion mutations c.451_453delTCC(p.Ser151del) and c.1121_1132del12 (p.Val374_Ala377del), one splicing mutation c.483 + 2 T > A, and an indel (deletion/insertion) mutation c.169_170delATinsG, which resulted in Methionine (Met) residue in position 57 changing to Glycine (Gly) with a premature termination signal (p. Met57GlyfsX29). All family members with diabetes or impaired fasting glucose as shown in Table 3, were genetically investigated and showed the same mutation as the proband. Four of the nine mutations were inherited from the father while five were from the mother. No mutation was found among the normoglycaemic relatives of the proband.

**Table 4:** Glucokinase (*GCK*) gene mutations in 9 patients and their family members from South China

Patient	Type of mutation	Location	cDNAmutation	Amino acid change	Domain localization/Secondary structure	Described
Case1/ Father	Missense	Exon 5	c.544G > A	p.Val182Met	Small domain/α4 helix	Previously [14]
Case2/Father/Grandmother	Splicing	Intron 4	c.483 + 2 T > A			This study
Case3/Mother	Deletion	Exon 9	c.1121_1132del12	p.Val374_Ala377del	Large domain/α11helix	This study
Case 4/Mother	Deletion	Exon 4	c.451_453delTCC	p.Ser151del	Small domain/ loop	This study
Case 5/Mother	Splicing	Intron 6	c.679 + 1G > A			Previously [15]
Case 6/Mother/ Grandfather	Indels	Exon 2	c.169_170delATinsG	p. Met57GlyfsX29	Large domain/β-strand 1	This study
Case 7//Father	Missense	Exon 8	c.883G > A	p.Gly295Ser	Large domain/α12 helix	Previously [4]
Case 8/Mother/Grandmother	Missense	Exon 5	C.572G > A	p.Arg191Gln	Small domain/α4 helix	Previously [4]
Case 9/Father/Grandfather	Missense	Exon 2	c.122 T > C	P.Met41Thr	Large domain/α2 helix	Previously [4]

## Discussion

*GCK* mutation is a common cause of incidental hyperglycaemia in otherwise well asymptomatic children in other populations [16, 17]. We found *GCK* mutations in nine out of eleven children with asymptomatic hyperglycaemia in our hospital cohort. There is a recent report from another children’s hospital in China, where three families with genetically confirmed MODY2 were diagnosed in over a year, two probands were children with asymptomatic hyperglycaemia and had abnormal OGTT [18]. These observations suggest that *GCK* mutations may be a common cause of asymptomatic hyperglycaemia in Chinese children. Further study with larger cohort numbers is needed to confirm this.

The clinical features of our cohort in terms of glycaemic profile are very similar to other populations. The fasting glucose of 6.1–8.5 mmol/L and HbA1c 5.2–6.7% fell within the typical ranges reported in GCK-MODY. Stability in both parameters on follow-up for up to 5 years without drug treatment is consistent with GCK-MODY. Most GCK-MODY patients have a small increment in blood glucose (< 3.0 mmol/L) during an oral glucose tolerance test [11]. In this study, 2 out of the 9 cases had 2-h glucose increment > 3 mmol/L (3.5 mmol/L and 4.6 mmol/L respectively) on repeat OGTT at follow-up. Glycosuria was absent in all patients, reflecting the mild hyperglycaemia below renal threshold, similar to findings in GCK-MODY in Caucasian populations [2].

Neonatal diabetes mellitus, a monogenic disorder, needs to be considered in hyperglycaemia within the first 6 months of life. Insulin treatment is required and sulphonylurea is the treatment of choice if there are activating mutations in the *KCNJ11* or *ABCC8* genes [19]. In case 1 and case 3, neonatal diabetes mellitus was ruled out based on the clinical course of stable hyperglycaemia without pharmacotherapy. In case 2, the genetic analysis clinched the diagnosis of GCK-MODY rather than neonatal diabetes and drug treatment was confidently stopped. The detection of hyperglycaemia in early infancy that remains stable, as described in three cases (cases 1–3) here, is consistent with previous reports of hyperglycaemia being present early in life, including the neonatal period, in GCK-MODY [20]. Type 1 diabetes mellitus, the most prevalent form of diabetes in childhood, was ruled out in cases 4–9 as there was no ketosis without insulin therapy and pancreatic autoantibodies were negative. They (cases 4–9) were unlikely to have type 2 diabetes, the next common form of paediatric diabetes, as they lacked the typical features of obesity and acanthosis nigricans. However, the prevalence of type 2 diabetes is high in Asia and many Asian children with type 2 diabetes are not obese [21]. Incidental hyperglycaemia in an asymptomatic Asian child will always raise the possibility of development of type 2 diabetes especially if there is a positive family history. Differentiating GCK-MODY and pre-diabetic phase of type 2 diabetes in Asian children based on clinical features is therefore challenging. Genetic analysis for GCK mutation is vital to differentiate GCK-MODY from the pre-diabetic phase of type 2 diabetes in non-obese Asian children.

To date, more than 600 mutations of the *GCK* gene have been documented (Human Gene Mutation Database at the Institute of Medical Genetics in Cardiff: GCK Gene: http://www.hgmd.cf.ac.uk). Here we reported 9 different mutations, five of them (p.Val182Met, c.679 + 1G > A, p.Gly295Ser, p.Arg191Gln, and p.Met41Thr) have been previously reported, but not in a Chinese population; four mutations were novel, c.483 + 2 T > A, p.Ser151del, p. Met57GlyfsX29 and p.Val374_Ala377del. Human GCK is an allosteric enzyme consisting of two domains, the hexokinase small domain and the hexokinase large domain [22]. In this study the mutations were distributed evenly throughout the GCK protein: 4/9 (44.4%) in the small domain, 5/9 (55.6%) in the large domain. These findings are in agreement with studies from Caucasian populations which indicated no hot spot mutations were found in the GCK gene [4]. All of the mutations in GCK co-segregated with diabetes in more than one affected member of the family and none of the mutations was found among non-diabetic relatives of the proband. They are likely to have a damaging effect on GCK enzyme function. According to the Human Genome Variation Society (HGVS), splice site mutations that alter highly conserved nucleotides (position + 1, + 2, −1 and −2) can be predicted to affect the primary transcript or protein synthesis. Therefore the novel mutation c.483 + 2 T > A which caused T to A transversion at the 3′ donor splice site of exon 4 is likely a disease causing mutation. The indel mutation c.169_170delATinsG (p. Met57GlyfsX29) is predicted to be highly pathogenic, as it gives rise to a premature truncation of GCK protein at position 86. The deletion mutation c.1121_1132del12 (p.Val374_Ala377del) found in exon 9 is due to slipped mispairing during DNA replication, caused by the same dinucleotide CG on both sides of the deletion. However, functional study of the novel mutations on enzyme activity was not performed. Additional kinetic assays are necessary to establish the actual functional basis for the disease.

GCK-MODY is a condition of altered physiological set point of glucose homeostasis due to the mutation, rather than a pathological disease state. It requires no drug treatment, except during pregnancy if there is excessive fetal growth, as pharmacotherapy is ineffective in reducing blood glucose or HbA1c [2]. There is no increased risk of long-term diabetes-related microvascular and macrovascular complications [23]. Children with genetically confirmed GCK-MODY do not require regular follow-up as they are expected to have lifelong mild stable fasting hyperglycaemia without any known long-term adverse effects. However, they have the same risk of developing type 1 and type 2 diabetes as the general population [2]. They need to be reassessed and investigated for type 1 or type 2 diabetes if new suggestive clinical features arise.

Genetic confirmation of GCK-MODY will help predict clinical course and long-term prognosis, aid treatment and follow-up decisions. This is aptly illustrated by case 2 whereby unnecessary anti-diabetic drug treatment was stopped when GCK-MODY was confirmed genetically. Treatment cost can be reduced as unnecessary long-term use of medication and regular investigations as part of routine care of diabetes are prevented. There is a positive impact on the child and their family as frequent hospital clinic visits and blood taking for investigations for diabetes care are stopped. The parents can be reassured that there is no risk of long-term diabetes-related complications in their child. On the contrary, the other two children who have tested negative for *GCK* gene mutation are likely to have other types of diabetes, such as type 2 diabetes; and require closer monitoring and follow-up. When a child is known to have a *GCK* gene mutation, genetic screening for the *GCK* gene mutation can be offered to other family members with a diagnosis of diabetes; eliminating unneccessary anti-diabetic treament if a GCK mutation is confirmed.

## Conclusions

GCK gene mutations are detected in Chinese children and their family members with typical clinical features of GCK-MODY as described in other populations. GCK mutations are not as rare in the Chinese population as previously thought. Four novel mutations in the GCK gene were identified in our cohort of 11 patients.

## Abbreviations

FPG: Fasting plasma glucose
GAD: Anti-glutamic acid decarboxylase
GCK: Glucokinase
GCK-MODY: Glucokinase-maturity-onset diabetes of the young
IAA: Anti-insulin
ICA: Anti-islet cell
MODY: Maturity-onset diabetes of the young
NGS: Next-generation sequencing
OGTT: Oral glucose tolerance test
PCR: Polymerase chain reaction
